# Production- and Purification-Relevant Properties of Human and Murine Cytomegalovirus

**DOI:** 10.3390/v13122481

**Published:** 2021-12-10

**Authors:** Sanda Ravlić, Marija Brgles, Lea Hiršl, Stipan Jonjić, Beata Halassy

**Affiliations:** 1 Centre for Research and Knowledge Transfer in Biotechnology, University of Zagreb, 10000 Zagreb, Croatia; mbrgles@gmail.com (M.B.); bhalassy@unizg.hr (B.H.); 2 Center of Excellence for Viral Immunology and Vaccines, CERVirVac, 10000 Zagreb, Croatia; lea.hirsl@medri.uniri.hr (L.H.); stipan.jonjic@medri.uniri.hr (S.J.); 3 Croatia Center for Proteomics, Faculty of Medicine, University of Rijeka, 51000 Rijeka, Croatia

**Keywords:** HCMV, MCMV, purification, ion exchange chromatography, virus, ultracentrifugation, clarification, cytomegalovirus

## Abstract

There is a large unmet need for a prophylactic vaccine against human cytomegalovirus (HCMV) to combat the ubiquitous infection that is ongoing with this pathogen. A vaccination against HCMV could protect immunocompromised patients and prevent birth defects caused by congenital HCMV infections. Moreover, cytomegalovirus (CMV) has a number of features that make it a very interesting vector platform for gene therapy. In both cases, preparation of a highly purified virus is a prerequisite for safe and effective application. Murine CMV (MCMV) is by far the most studied model for HCMV infections with regard to the principles that govern the immune surveillance of CMVs. Knowledge transfer from MCMV and mice to HCMV and humans could be facilitated by better understanding and characterization of the biological and biophysical properties of both viruses. We carried out a detailed investigation of HCMV and MCMV growth kinetics as well as stability under the influence of clarification and different storage conditions. Further, we investigated the possibilities to concentrate and purify both viruses by ultracentrifugation and ion-exchange chromatography. Defective enveloped particles were not separately analyzed; however, the behavior of exosomes was examined during all experiments. The effectiveness of procedures was monitored using CCID_50_ assay, Nanoparticle tracking analysis, ELISA for host cell proteins, and quantitative PCR for host cell DNA. MCMV generally proved to be more robust in handling. Despite its greater sensitivity, HCMV was efficiently (100% recovery) purified and concentrated by anion-exchange chromatography using QA monolithic support. The majority of the host genomic DNA as well as most of the host cell proteins were removed by this procedure.

## 1. Introduction

Cytomegaloviruses (CMVs) are enveloped dsDNA prototypes of the β subfamily of *Herpesviridae*. Their genome of approximately 235 kb encodes ≈ 165 open reading frames [1] and places CMVs among mammalian viruses with the largest coding capacity [2]. Human CMV (HCMV) is a ubiquitous virus that establishes a systemic latent/permanent infection with a seroprevalence of 50% to 100% in the general adult population. In immunocompromised patients, organ transplant recipients, and congenitally infected infants HCMV can cause severe disease [3,4,5,6,7,8], and for this reason a vaccine against HCMV is of great importance. Infection in healthy individuals is typically asymptomatic and is characterized by incredibly strong immune responses, including the accumulation of a very large, viable T cell population [9,10,11,12,13,14,15]. There is a strong interest in CMV as an engineered vector for vaccination against other viral diseases and as “live drugs”, including therapeutic oncolytic agents [16,17,18,19,20,21,22,23,24,25,26,27,28,29,30]. This great attention is based on the size and engineering flexibility of the CMV genome [23] along with its superinfection capacity [5], which goes beyond pre-existing immunity [24]. The broad spectrum of potential applications of CMVs as vector platforms came from the studies on animal models and, although recombinant HCMV expressing heterologous antigens have never been clinically tested, several attenuated HCMV vaccine candidates have entered early clinical trials as vaccines against HCMV [31,32]. However, there are no licensed CMV vaccines and the publications on their pharmaceutical development are limited; therefore, great efforts are being made to better understand the virus [33].

In addition, regulatory expectations tend to make vaccine manufacture purer and better characterized, in the direction of cell culture-based production that minimizes cross contamination or allergy reactions (ref). The use of serum free media for viral production dramatically decreases the amount of contaminating proteins (bovine serum albumin, transferrin, and immunoglobulins) and lipids, but reports demonstrating successful viral production in serum free media are scarce [34,35,36]. Quality and quantity of virus production highly depends on the parameters of upstream and downstream processes. Establishment of a process that results in the production of high titer virus yields is the goal of upstream process development. Once the peak in the virus production has been reached, the downstream processes begin by harvesting the culture. To reduce a burden from the upstream processing, the clarification step primarily removes whole cells, cell debris, colloids, and large aggregates, and like any other purification step it needs to be optimized to achieve maximal product yield and purity [37]. Low speed centrifugation as a choice for clarification removes cells and cell debris by sedimentation. Membrane filters retain particles by size exclusion and do not have a high ability to retain impurities, which mostly depends on the cell culture conditions such as cell density or cell viability at harvest [38]. Membranes with cut-offs in the range of 0.1–0.65 µm have been successfully used to retain cells, cell debris, and other large contaminants [39,40]. 

Furthermore, it is obligatory to perform stability studies of the virus and/or viral vector in answer to the environmental conditions to which the virus will be exposed during purification procedures [41]. From the downstream processing point of view, virus instability is translated into low overall recoveries after the purification steps. It is very rational to select the most convenient storage temperature as well as stabilization media in order to store viral stocks, because freeze and thaw cycles greatly affect viral infectivity [42]. 

Ultracentrifugation (UC) is a well-known and established classical method for the concentration and purification of viruses on a laboratory scale. By using UC, a 100-fold or over concentration of viral particles can easily be achieved. However, the infectivity of concentrated viral stocks usually does not increase proportionally with the concentration factor, and often the infectivity does not increase at all. The reason for infectivity loss after the UC is associated with extended processing time and the shear forces imposed on the virus particles [43].

Advances in the development of chromatographic columns suitable for large and fragile virus structures have enabled efficient virus purification. One of the most important chromatographic matrices for virus purification are monolithic columns due to their special characteristics: very high porosity, high binding capacity for very large molecules, and mass transport based on convection [44]. There are reports that monoliths with large channels of 6 µm are much more suitable for viruses than those with 1.5 µm, although the diameter of viruses is well below 1.5 µm [45,46]. All modes of chromatography can be used for the purification of viruses [47], but ion-exchange [47,48,49], hydrophobic interaction [46,50], and affinity chromatography [45,51] have gained most interest due to the simplicity and power of separation. 

As well as the process related impurities (cell culture reagents, additives, purification process substrate), great attention should also be paid to product related impurities, e.g., virus aggregates, incorrectly structured particles, host cell protein, and DNA residues [52]. One of the impurities originating from the host cell culture are exosomes. Exosomes are vesicles, containing proteins and nucleic acids, produced by all cells for communication. Exosomes are structurally very similar to enveloped viruses, and in cell cultures infected by viruses exosomes can even share some of the viral proteins and nucleic acids due to the similar pathways of biogenesis in the cell [53], thus making their separation and analytical monitoring very complicated. In addition to viral particles and exosomes, infected cells also produce non-infectious enveloped particles (NIEPs) and capsidless dense bodies (DB) that egress in parallel with viral particles and are comparable to them in surface protein composition [54,55].

The aim of our paper was to develop a small-scale manufacturing process for HCMV, and also to comparatively investigate HCMV and MCMV according to different typical upstream and downstream processing steps. We carried out a detailed investigation of HCMV and MCMV growth kinetics in MRC-5 and M2-10B4 cells, respectively. Their stability was compared during storage in different conditions, and their robustness towards several manipulation procedures was investigated by the following: filtration and low speed centrifugation for clarification purposes, along with ultracentrifugation as a method for concentration and purification. Finally, we investigated possibilities to purify both viruses by ion-exchange chromatography. During the upstream and downstream processes, we also examined the behavior of exosomes, one of the impurities very hard to monitor and separate from viable virus suspension. To our knowledge, this is the first comprehensive study on the purification of human cytomegalovirus, and it is our hope that the information’s presented here benefits future developments.

## 2. Materials and Methods

### 2.1. Cell Cultures

The MRC-5 cell line was purchased from ECACC (ECACC 05012101). The M2-10B4 cell line was purchased from ATCC, CRL-1972 (Manassas, Virginia, United States). Cells were maintained in Minimal Essential Medium with Earle’s salts (Capricorn Scientific, Ebsdorfergrund, Germany, Cat-No: MEM-XA) supplemented with 2 mM L-Gln (Capricorn Scientific, Ebsdorfergrund, Germany, Cat-No: GLN-B), 100 IU/mL penicillin/100 µg/mL streptomycin (Capricorn Scientific, Ebsdorfergrund, Germany, Cat No: PS-B), and 10 % FBS (Capricorn Scientific, Ebsdorfergrund, Germany, Cat-No: FBS-12A), in a 5% CO_2_ environment at 37 °C. Passaging was performed every 3–4 days, and trypsin was removed by centrifugation (270× *g* for M2-10B4, 300× *g* for MRC-5, 5 min).

### 2.2. Virus and Exosomes Production

MRC-5 and M2-10B4 cells were infected with HCMV TB40/E strain and MCMV Smith strain, respectively. The infections were carried out in a suspension at a multiplicity of infection (MOI) of 0.1 and 0.01 for HCMV, and a tenfold increases of MOI from 0.0001 to 1 for MCMV. MRC-5 cells were seeded at 7.5 × 10^4^ cells/cm^2^, while M2-10B4 cells were seeded at 4 × 10^4^ cells/cm^2^. After 24 h of M2-10B4 infection with MCMV and 48 h of MRC-5 infection with HCMV, the infected cells were washed and left in the medium without FBS. For viral growth kinetics, supernatant samples were taken daily for all investigated MOIs until the end of the infection, stabilized with gelatin-based stabilizer (a proprietary formulation of the Institute of Immunology, Inc., Zagreb, Croatia) or 5% FBS, for HCMV and MCMV, respectively, and stored at −75 °C until analysis. For concentration and purification purposes, the first harvest of HCMV supernatant, infected with MOI 0.1, was carried out when the cytopathic effect became visible, with frequent follow-up harvests (at 8,12, and 16 days) until all cells were separated from the flask.

For MCMV concentration and purification experiments, the supernatant, infected with MOI 0.01, was harvested at the peak of viral infectivity, 5 days post infection (dpi). Clarification of the supernatant was performed by microfiltration using a syringe and a 0.45 μm PVDF filter (Millipore, France), preceded by low speed centrifugation (3220× *g*, 7 min) in cases where the cytopathic effect was strong and many cells separated from the flask. Exosomes were produced and clarified using the same procedures without an infection with the virus.

### 2.3. Infective Virus Quantification

Quantification of HCMV and MCMV infectious particles was performed using 50% cell culture infective dose (CCID_50_) assay on MRC-5 and M2-10B4 cells, respectively, as previously described [56]. Medium used throughout the test was MEM supplemented with 10% FBS for MRC-5 cells and RPMI 1640 (Capricorn Scientific, Ebsdorfergrund, Germany, Cat-No: RPMI-XA) supplemented with 5% FBS for M2-10B4 cells, both supplemented with 2 mM L-Gln, penicillin (100 IU/mL) and streptomycin (100 µg/mL). The CCID_50_ assay lasted for 14 and 7 days, for HCMV and MCMV, respectively. The dependence of viral reference sample titer on cell population doubling level (PDL) was also examined.

### 2.4. Total Particle Quantification and Size Determination

Quantification of total particles was performed using a NanoSight LM10 instrument equipped with an sCMOS camera, and with a red laser (Malvern Panalytical Ltd, Malvern, UK). Nanoparticle Tracking Analysis (NTA) was performed on samples that were diluted to obtain 10–100 particles in the field of view. Each sample was measured three times, and 60 s videos of particles under Brownian motion were obtained with the camera level fixed at 10 and analyzed with detection threshold 5 using NTA 3.4 software. For particle size comparison, three diameter parameters were used: mean, mode, and D90 value, which denotes that 90% of particles have the indicated diameter or smaller. The ratios of the mean, mode, and D90 of the examined sample to the starting sample were determined, and the average value of these three ratios was taken as the size percentage of the sample in question. The recovery of all particles in the sample (both infectious and noninfectious) was expressed as the percentage of particles found in the experimental sample compared to particles found in the starting sample.

### 2.5. Storage Stability Study

A storage stability study was performed with virus preparations formulated with or without stabilizers: gelatin-based stabilizer (Institute of Immunology, Inc., Zagreb, Croatia) and 5% FBS (Capricorn, Ebsdorfergrund, Germany). All formulations were stored at 4 °C, −20 °C, and −75 °C and analyzed by CCID_50_ assay in a time dependent manner. The results were compared to those obtained in the starting virus sample subjected to CCID_50_ assay immediately after harvest.

### 2.6. Ultracentrifugation of Viruses

Ultracentrifugation (UC) of viruses was performed in a Beckmann Coulter preparative ultracentrifuge using an SW28 rotor with polyallomer cuvettes at 141,000× *g* for 1, 2, and 4 h, respectively. Supernatant aliquots were collected for subsequent analysis, while the pellets were resuspended in PBS. Infective viruses and total particles were quantified in initial suspension subjected to UC as well as in the supernatant and resuspended pellets after UC, and they were used to express recovery (%). Particle size changes were also monitored by NTA.

### 2.7. Chromatography

Chromatography was performed using ÄKTA pure M25 (General Electric Company, Boston, MA, United states). Samples were loaded using a sample pump at a flow rate of 5 mL/min. For ion-exchange chromatography, QA and SO_3_ columns were used. All columns were 1 mL column volume and 6 µm channel diameter, purchased from BIASeparations (Ajdovščina, Slovenia, EU). The binding buffer in ion-exchange chromatography was 50 mM MES, pH 7.3 or 50 mM MES, pH 7.3, 0.15 M NaCl, and elution was performed using a stepwise gradient of NaCl. All buffers were filtered through a 0.45 µm filter. Each chromatographic fraction was assayed by NTA, and samples for CCID_50_ assay were stabilized immediately (gelatin-based stabilizer for HCMV, 5% FBS for MCMV) and stored at −75 °C until analyzed.

### 2.8. ELISA Quantification of MRC-5 Host Cell Proteins

Host cell proteins were quantified using sandwich ELISA, as previously described [43], with antibodies produced in-house directed against MRC-5 proteins.

### 2.9. PCR Detection of Genomic DNA

Genomic DNA was extracted from MRC-5 and M2-10B4 cells, as were chromatographic samples using the classic phenol-chloroform method, as described before [57]. The concentration and purity of the extracted DNA was analyzed with both spectrophotometer (Multiskan^®^ Spectrum spectrophotometer, Thermo Scientific, Waltham, MA, USA) and fluorometer (Quantus™, Promega Corporation, Madison, WI, USA). To calculate the quantity of host cell DNA in chromatographic samples, six-point DNA standard curves, ranging in concentration from 10^−3^ to 10^2^ ng/µL, were generated from serial dilutions of MRC-5 and M2-10B4 genomic DNA. After DNA isolation, each sample was analyzed in triplicate by qPCR in a 96-well optical reaction plate. In the same plate, the DNA standards for the standard curve, and negative controls, were each analyzed in triplicate wells. qPCR was performed with an Applied Biosystems 7500 Real-Time PCR system (Life Technologies, Forster City, CA, USA) using the following thermal cycling conditions: initial heat denaturation at 50 °C for 2 min and 95 °C for 10 min, followed by 40 cycles each of 95 °C for 15 s and 60 °C for 1 min. An amount of 9 µL of sample genomic DNA was amplified in a total volume of 20 µL mixture containing 2 × TaqMan Universal PCR Master Mix and TaqMan gene expression assay for human beta actin gene and mouse beta actin gene, according to the manufacturer’s protocol (Thermo Fisher Scientific, Waltham, MA, USA).

Standard curve plots were generated from instrument export data: log DNA values versus Ct values. Standard curve statistics, including slope, y intercept, R2, and % efficiency were determined using Excel 2019. Unknown DNA values were extrapolated from the standard curve y = mx + b, from which follows “extrapolated unknown” = 10 (Ct-y-intercept)/slope. 

## 3. Results

### 3.1. Viral Growth Kinetics In Vitro

**Figure 1:**
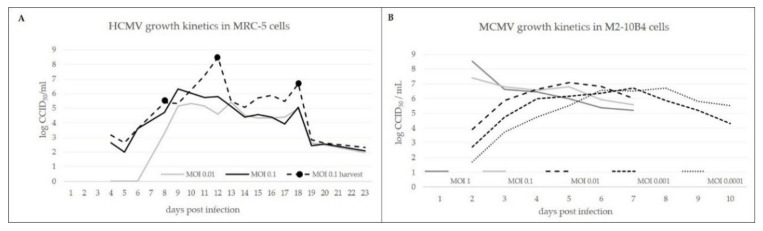
Viral growth kinetics in vitro. (**A**) HCMV growth kinetics in MRC-5 cells. (**B**) MCMV growth kinetics in M2-10B4 cells.

In order to follow the viral growth kinetics in vitro, we harvested the extracellular viruses from the virus-infected MRC-5 and M2-10B4 cell culture supernatants at various time points post infection and determined infectious virus concentrations (Figure 1A,B). Our studies showed that HCMV grows more slowly on MRC-5 cells than MCMV on M2-10B4 cells and that the highest HCMV infectivity can be expected after the eighth day, post infection (dpi) with all investigated MOIs. A good feature of HCMV growth on MRC-5 cells is that the virus can be repeatedly harvested to yield higher amounts of infective particles, as shown in Figure 1A, which is not the case with MCMV growth on M2-10B4 cells because the cytopathic effect strongly destroys the cells. MCMV showed various growth kinetics depending on the different MOI applied. An MOI of 1 and 0.1 gave the highest MCMV infectivity on 2 dpi, while the smaller MOIs (0.01–0.0001) resulted in the highest viral infectivity on 5 dpi (Figure 1B). 

**Figure 2:**
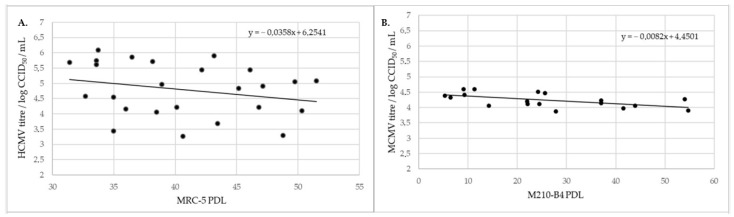
Reference virus sample titer relative to PDL. (**A**) HCMV, (**B**) MCMV.

Cell aging, expressed as the cells’ population doubling level (PDL), slightly impacted the virus titer, as demonstrated by lower viral titer in cells of higher PDL, which can be observed for both viruses, HCMV and MCMV (Figure 2). It is also evident that the CCID_50_ assay provides greater uniformity of MCMV reference titers than of HCMV titers, which we attribute to the duration of the CCID_50_ assay, which lasts for 7 and 14 days, respectively.

### 3.2. Impact of Clarification on Viral Samples 

Due to cytopathic effect, the viral suspension harvested from the virus-infected cell culture needs to be purified from cell parts. This removal is obligatory, either for further processing or for biophysical measurements. Consequently, we investigated the effect of filtration through a 0.45 μm PVDF membrane, which, in the case of MCMV, was preceded by low-speed centrifugation (3220× *g*, 7 min) due to a strong cytopathic effect. Particle size, total particle recoveries, and infectivity recoveries of all samples were calculated relative to the data obtained for the starting sample (Table 1). The results showed that particle size and total particle recoveries were not affected by filtration and centrifugation. However, infectivity loss was detectable, being more pronounced for HCMV (cca 25%) than for MCMV (cca 20%). Examination of MRC-5 and M2-10B4 exosomes has shown that they were slightly smaller compared to viral samples in terms of particle size, and they were not affected by filtration.

### 3.3. HCMV and MCMV Storage Stability

**Figure 3:**
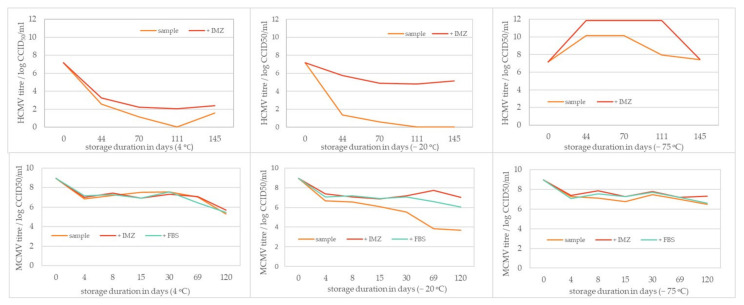
HCMV (upper row) and MCMV (lower row) viral infectivity titer after the sample storage with/or without stabilizer (gelatin-based stabilizer and FBS) at three different storage temperatures (+4 °C, −20 °C, and −75 °C) and extended time of duration. “Sample” signifies the viral suspension itself stored without stabilizers. “+IMZ” indicates the addition of 70% gelatin-based stabilizer, and “+FBS” indicates the addition of 5% FBS as stabilizer.

The ability of two types of excipients to stabilize HCMV and MCMV against prolonged storage at different temperatures was examined, along with the stability of viral samples stored without the stabilizers. The effect of stabilizers (gelatin-based or FBS) depended largely on the storage temperature, with the overall best preservation of viral infectivity found at −75 °C, for both viruses. Furthermore, the HCMV and MCMV titer decreased with prolonged storage time, which is best seen in the samples stored at 4 °C and −20 °C. Storage of HCMV samples without any stabilizer, at 4 °C and −20 °C, gave poor recovery of virus infectivity and was determined to be insufficiently optimal because ≥4 log loss in viral titer was observed after any storage time. Similar results were observed for MCMV stored at −20 °C, with a loss in viral infectivity of ≥2 log. In both cases, the use of stabilizers preserved viral infectivity: gelatin-based stabilizer for HCMV (Figure 2, + IMZ), and gelatin-based and FBS for MCMV (Figure 2, +IMZ, +FBS). The use of stabilizers had no impact on MCMV infectivity loss for samples stored at 4 °C, meaning that the thawing and/or temperature changes of stored MCMV samples had the biggest impact on virus infectivity loss. The use of gelatin-based stabilizer and 5% FBS yielded satisfactory results at −75 °C for both MCMV and HCMV. MCMV showed greater robustness, resulting in a more uniform loss of infectivity during the time of investigation (Figure 3).

The starting rise in titer for samples stored at −75 °C is most probably due to incorrect determination of the titer in the initial sample. It is highly probable that it was higher. In that case, the drop in titers during storage at +4 °C and −20 °C would be even more pronounced, while storage at −75 °C would prove useful for this virus. The infective HCMV quantification was the most challenging task in our work, and further investigation is needed to improve its performance.

### 3.4. Ultracentrifugation

Ultracentrifugation (UC) is a widely used method for laboratory-scale virus purification and concentration. We examined how the duration of UC (1,2,4 h) influences the virus infectivity, particle size, and total particle recoveries. HCMV was extremely sensitive to ultracentrifugation, with more than 90% infectivity loss under all investigated conditions (Table 2). The best HCMV infectivity recovery was observed in the pellet fraction after 2 h of ultracentrifugation and amounted to a little less than 8%. In contrast, MCMV was successfully concentrated by 1 h ultracentrifugation, with no loss of infectivity (Table 2). Prolonged ultracentrifugation times significantly reduced infectivity. Loss of infectivity was partially due to the loss of total particles, indicating that the shear forces required for pelleting the virus also cause a large decay of viral particles. Effectiveness of the UC proved to be better for MCMV in all investigated time periods, yielding a particularly high recovery of virus infectivity.

### 3.5. Ion-Exchange Chromatography

**Figure 4:**
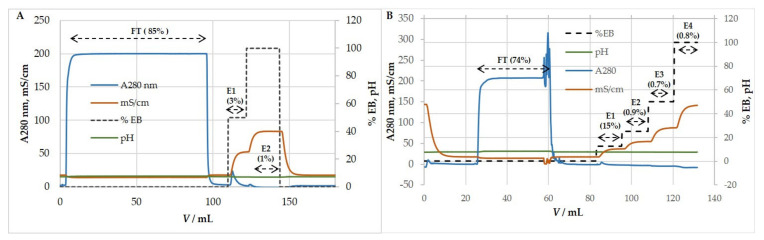
Representative (**A**) HCMV and (**B**) MCMV ion-exchange chromatographic profile performed using a cation exchanger, SO_3_ monolith column. HCMV; Binding buffer: 50 mM MES, pH 7.3, 0.15 M NaCl. Elution buffer: 50 mM MES, pH 7.3, 1 M NaCl. MCMV; Binding buffer: 50 mM MES, pH 7.3, 0.15 M NaCl. Elution buffer: 50 mM MES, pH 7.3, 2 M NaCl. Percentage of total particles is denoted with each corresponding fraction. FT—flow-through, E—eluate.

Purification of HCMV and MCMV by ion-exchange chromatography was tested in cation- and anion-exchange mode using strong exchangers. The efficiency of purification was estimated by quantifying infective (CCID_50_) and total particles (NTA) in all chromatographic fractions and comparing them to the ones in the loading sample. When optimal chromatographic conditions for virus purification were set up, analyses were extended to host cell DNA and protein content in all fractions. Our investigations showed that both HCMV and MCMV bind to the QA column and bind only very slightly to the SO_3_ column (Figure 4). Results showed that less than 5% and 18% of infective HCMV and MCMV particles and total particles bound to SO_3_ column, respectively, while most of the particles passed through the column. Therefore, ion-exchange experiments were focused on anion-exchange mode. 

#### 3.5.1. Anion-Exchange Chromatography for Purification and Concentration of HCMV 

**Figure 5:**
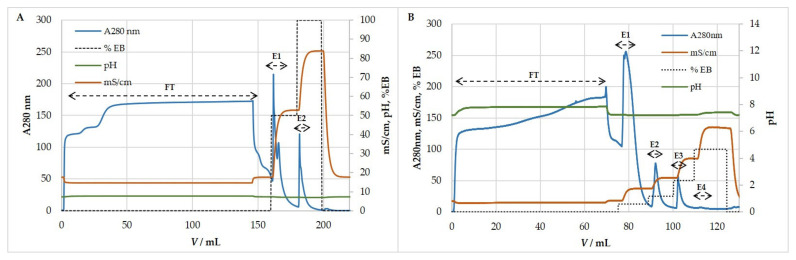
Step gradient elution profile of HCMV infected cell supernatant using an anion exchanger, QA monolith column. (**A**) Binding buffer: 50 mM MES, pH 7.3, 0.15 M NaCl. Elution buffer: 50 mM MES, pH 7.3, 1 M NaCl. Infective viruses eluted in both peaks as showed in Table 3. (**B**) Binding buffer: 50 mM MES, pH 7.3. Elution buffer: 50 mM MES, pH 7.3, 2 M NaCl. Most of host cell proteins did not bind to the column. Infective viruses eluted in the first peak using 0.25 M NaCl. The majority of host cell DNA eluted in the second peak using 0.5 M NaCl. FT—flow-through, E—eluate.

Preliminary experiments using the QA column were performed with a binding buffer containing 0.15 M NaCl and elution by a two-step increase in ionic strength, 0.57 and 1 M NaCl (Figure 5A). Results showed that all of the HCMV binds to the column and is successfully removed by elution with salts, with an overall high yield of recovered viral infectivity (~100%) (Table 3). When the number of total particles in the load exceeded 6 × 10^11^, particles were also found in the flow-through fraction (FT).

Further optimization of the purification process generated the best chromatographic conditions: binding buffer without NaCl and elution performed in four steps; 0.25 M, 0.5 M, 1 M, and 2 M NaCl (Figure 5B). Most of the infective HCMV repeatedly eluted with 0.25 M NaCl. Recoveries of infective HCMV were very high (Table 4). Most of the host cell proteins were detected in the flow-through fraction, thus being separated from the virus, which was retained on the column. The majority of the host cell DNA was bound to the anion-exchanger as expected and eluted with 0.5 M NaCl (Table 4), implying a higher negative charge of DNA than HCMV particles. Overall, we achieved efficient separation of these two contaminants from concentrated HCMV suspension.

#### 3.5.2. Anion-Exchange Chromatography for Purification and Concentration of MCMV

**Figure 6:**
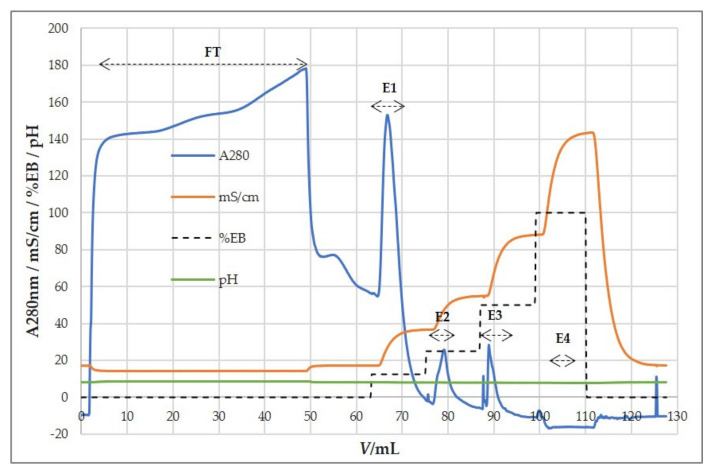
Step gradient elution profile of MCMV infected cell supernatant using an anion exchanger, QA monolith column. Binding buffer: 50 mM MES, pH 7.3, 0.15 M NaCl. Elution buffer: 50 mM MES, pH 7.3, 2 M NaCl. Infective viruses eluted in first peak using 0.32 M NaCl. FT—flow-through, E—eluate.

MCMV behaved similarly to HCMV in anion-exchange chromatography. Experiments on purification and concentration of MCMV were performed using binding buffer containing 50 mM MES, pH 7.3, 0.15 M NaCl, while elution was achieved by four-step increases in ionic strength: 0.32 M, 0.61 M, 1.075 M, and 2 M NaCl containing elution buffer (Figure 6). Most of the infective MCMV eluted with 0.32 M NaCl, and during the eight measurements, the elution profile was repeatable (Table 5). Preliminary studies proved that the majority of host cell DNA eluted with 0.61 M NaCl (results not shown), which also resembled the chromatography results of HCMV. 

### 3.6. Exosomes

**Figure 7:**
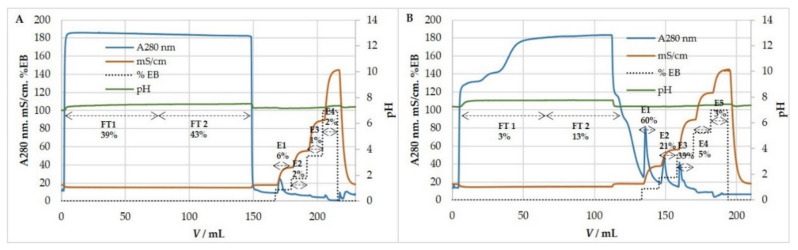
Representative MRC-5 exosome ion-exchange chromatographic profile performed using (**A**) cation-exchange on a SO_3_ column (total particle load is 2.36 × 10^11^), (**B**) anion-exchange on a QA column (total particle load is 1.73 × 10^11^). Percentage of total particles is denoted with each corresponding fraction. FT—Flow-through, E—eluate.

Because exosomes are very difficult to discern from virus particles, we wanted to evaluate the ion-exchange chromatography of exosomes to be able to assess possible contamination of HCMV in chromatography under the same conditions. Figure 7 shows chromatography of exosomes derived from MRC-5 cells on SO_3_ and QA monolithic columns. Results indicate that only a small fraction of exosomes binds to the SO_3_ column, and in contrast a large portion of exosomes binds to the QA column, all of which resemble the chromatography results of HCMV. Exosomes bound to the QA column eluted mostly up to 1 M NaCl.

## 4. Discussion

The goal of our study was to establish a small-scale laboratory production of pure and concentrated HCMV. We investigated the robustness of HCMV towards different upstream and downstream processing steps and compared it to MCMV, a model virus for developing CMV-based vaccines and gene vectors.

The production of high titer yields of HCMV and MCMV was achieved by infections in a suspension using 10% serum to allow easier adhesion for MRC-5 and M2-10B4 cells. After 24 h for MCMV infection and 48 h for HCMV infection, serum was removed, and virus growth was enabled in serum-free media to maximally reduce protein content. The transition to serum-free media or even completely animal-component-free system is a major step forward in the production of vaccines, e.g., lower cost, reduced risk of contamination, and a cleaner product recovery [35,58]. HCMV was grown on fibroblast-like human diploid cells, MRC-5, which have been used as a common cell substrate for vaccine production of varicella zoster virus, MMR, polio, rotavirus, rabies, hepatitis A, and dengue virus [59,60,61,62]. In agreement with previously published viral kinetics, HCMV infection of MRC-5 cells with low MOI resulted in an infectivity peak at day 8, post infection [63,64,65]. At the same time, MCMV, having a shorter viral cycle in M2-10B4 cells, reached the infectivity peak earlier, at day 2 or 5 post infection, depending on the MOI applied, which is in accordance with the investigation of Zurbach et al. [66].

After reaching a peak in virus production, a prerequisite for downstream processing is the removal of host cell parts and large aggregates, which is usually done by low-speed centrifugation or filtration [41]. Filtration of HCMV did not affect the size of particles nor the total particle count; however, HCMV infectivity was reduced for 25%. A reduction in virus titer during filtration probably results from the stress imposed on the virus, such as pressure, membrane fouling, or mechanical disruption due to passage through filter pores obstructed with cell debris [67]. Due to the fact that no change in particle size or concentration was observed between crude and clarified HCMV samples, we can conclude that the removal of larger virus particles or virus aggregates from the crude sample did not contribute to the reduction of infectivity. For the purpose of MCMV clarification, the filtration process was not satisfactory due to filter clogging, so we had to perform the low speed centrifugation step that preceded the filtration. Filter capacity highly depends on the cell culture conditions, such as cell density or cell viability at harvest [38]. These parameters influence the amount of cell debris and large aggregates that can plug depth filters and membranes, leading to reduced capacities [68]. In the case of MCMV growth on M2-10B4 cells, the cell viability at harvest was very poor due to cell growth in serum free conditions (results not shown), and cell debris was large. The results of particle concentration measurements implied the slight reduction in MCMV total particle count, while CCID_50_ assay showed 20% loss of infectivity. Reduction in virus titer during centrifugation might be caused by the shear stress imposed by the centrifugal force or even by the partial pelleting of virus aggregates [67]. The clarification results showed that exosomes produced by MRC-5 and M2-10B4 cells were slightly smaller in size than the particles measured in the HCMV and MCMV harvests, respectively, and the clarification processes did not affect the size or concentration of the exosomes. Size determination of the filtered viruses using a Nano-Sight instrument revealed that the average diameters are within a previously reported range of approximately 200 nm, for both viruses [69,70].

Viruses and viral vectors are inherently unstable and infectivity titer losses can readily occur without defining appropriate stabilizer and storage conditions. Different additives can be used as stabilizers during virus storage to preserve virus infectivity, and it is also very important to know whether some compounds should be avoided due to destabilizing effects. Fetal bovine serum (FBS) and gelatin-based stabilizers were tested, along with samples of the crude virus. By examination of different temperatures and storage lengths, a gelatin-based stabilizer and FBS showed the highest stabilization capacity at temperatures of −75 °C. It was observed that HCMV-based vectors were prone to accelerated titer loss upon freeze-thaw or storage at 2–8 °C [71], which was also the case in our research, regardless of the stabilizer used. The infectivity of crude MCMV samples stored at 4 °C did not show a large decrease during prolonged storage, which is very useful in basic research because there is no need for stabilization or freezing of the samples. 

In order to avoid inter-assay discrepancies, it is highly recommended to develop an in-house virus standard, which was delivered for both HCMV and MCMV. The titer of the viruses’ standards was used to monitor both assays’ reproducibility. While MCMV titration was quite reproducible, the HCMV titration results were more variable between runs. We attribute this to the slow growth of HCMV in MRC-5 cells requiring a prolonged period of time for stable cytopathic changes to develop in the cell layer. As a result, duration of HCMV titration is 14 days, during which period the cells do not obtain fresh nutrients.

In order to improve efficiencies of viral vaccines and vectors, it is required to produce scalable purification and concentration strategies to remove the contaminants present in the harvested supernatants while preserving the functionality of the virus. A widely used method for preparing highly purified viral material is equilibrium density ultracentrifugation in sucrose or sorbitol gradients, but these techniques are most suitable for studies that do not require preservation of viral activity [72,73]. Moreover, the high viscosity of sucrose has been associated with loss of surface protein structures and thus loss of infectivity upon purification [41]. For the reasons stated, we opted for virus pelleting by one step ultracentrifugation, as is traditionally employed to concentrate viruses and where a high concentration of the virus stocks (over 100-fold) can easily be attained by resuspending viral pellets in small volumes of resuspension buffer. In most cases, the infectivity of the virus does not increase proportionally with the concentration factor, which was the case in our investigation of HCMV ultracentrifugation at all investigated times. This effect of infectivity loss can be attributed to loss of active viral particles due to shear stress or extended processing time. On the other hand, MCMV was efficiently concentrated, with the restoration of its infectivity up to 100% by 1 h of UC.

Our approach to the purification and concentration of HCMV and MCMV based on ion exchange chromatography resulted in highly purified preparations from crude harvests with excellent recovery, without the need for enzymatic treatment. Results indicate that the anion exchange-based approach showed excellent retention of HCMV and MCMV particles, as well as exosomes, implying that all have an acidic pI at physiological conditions. HCMV and MRC-5 exosomes exhibited similar behavior and eluted mostly with 0.25 M NaCl and completely up to 1 M NaCl. Importantly, recoveries of HCMV infective particles were approaching 100%. Some variations among different chromatographic runs were probably present due to the variability of the HCMV CCID_50_ assay, but it is clear that there was an increase in the titer in the elution fraction, and it can be expected to be more uniform and even higher when scaling up. The low salt required for elution of the majority of infective HCMV means lower stress during elution. Interestingly, our previous work on the mumps and measles viruses derived from Vero cells showed high binding efficiencies to the QA column and very low binding to the SO_3_ column, but recoveries of infective particles were small, most probably due to the higher binding affinity and sensitivity to the higher salt amounts required for elution [74]. HCMV obviously exhibits surface properties, enabling the successful use of ion-exchange chromatography. Possibly, some influence on this is the difference of host cell lipids (Vero in difference to MRC-5) from which the HCMV and mumps are derived, resulting in differences in the lipid composition of viruses, in addition to virus surface specificities. MCMV showed similar behavior to HCMV in anion-exchange chromatography, although additional optimization is required in the case of MCMV purification.

NTA results indicate that particles in the HCMV chromatographic flow-through fraction were smaller in size than the starting sample (Table 3 and Table 4), and similar behavior has been previously observed with other viruses [74,75]. When this data is compared to the amount of infected virus particles, it can be concluded that these smaller particles are non-infective ones. However, experiments with loads below 6 × 10^11^ particles/mL had a small number of particles in flow-through, i.e., they bind to the column, but when there is competition with higher binding particles they end up in flow-through. In the MCMV chromatographic, flow-through fraction particles smaller in size compared to the starting sample were not detected, but as previously stated, additional process optimization is required. 

The important feature of the purification process is the removal of impurities. Limits for host cell proteins are not defined but should aim to be below 100 ppm [50], and for host cell DNA might depend on the type of host cells but are generally set to 10 ng/dose [51]. The results presented here show that the majority of host cell DNA was eluting after the peak containing most of the infective HCMV, meaning a higher negative charge density of DNA than HCMV and therefore a higher salt concentration required for DNA elution from an anion exchange column. Amounts of host cell DNA analyzed in all chromatographic fractions are very small and well below the limits. Additionally, most of the starting amount of host cell proteins was found in the flow-through fraction, and the concentration of host cell proteins in elution fractions containing most of the infective HCMV is roughly the same (Table 4). The appearance of host cell proteins in elution fractions does not seem favorable, but it might be the result of the fact that some viruses might contain host cell proteins incorporated in the virus particle. This might be the result of fortuitous incorporation of host cell proteins during budding or active incorporation into enveloped viruses. The presence of host cell proteins in virus particles was previously confirmed in HCMV [11] and other viruses [41,53,74,76,77]. However, there is always a question of the purity of virus particles being analyzed in these reports due to possible contamination by exosomes, which are very hard to discriminate from enveloped viruses [78]. Exosomes are very similar to virus particles in terms of size and density and can share the same proteins [53,78,79], but the presence of these proteins at the surface of exosomes and their function have not been studied extensively. Furthermore, HCMV-enveloped proteins, gB and gH, which are essential for HCMV infectivity, were found incorporated in the exosomes released by HCMV infected cells [80]. The particle size most commonly found in our HCMV infected harvest corresponds with the previously reported particle size of HCMV preparations and is around 200 nm, while the exosome size produced by MRC-5 cells is slightly lower, as also confirmed by previous research [80]. It has recently become evident that viral suspensions, in particular HIV-1, are in fact complex mixtures of virions and small exosomes released by both infected and uninfected cells [53,74,79]. However, the impact of exosomes, or their possible negative role in virus preparation for biomedical use, is still not clear, and major reason for this is the biophysical and chemical similarity of exosomes and enveloped viruses. Exosomes carrying HCMV viral surface proteins may contribute to various physiological effects in which HCMVs have been implicated because they should both target the same cells expressing HCMV receptors [80]. By sharing the same biogenesis, pathway exosomes acquire viral proteins and, as a result, viral stock is composed of exosomes, defective enveloped particles, and infectious virions [53,79]. The structure and protein composition of defective enveloped particles, NIEPs and DB, is comparable to that of virions, except for the presence of assembly protein and lack of DNA in NIEPs, and lack of viral capsids and DNA in DB [55]. Given the similarity in the protein surface composition of viruses and defective enveloped particles, we can assume a resemblance in their chromatographic behavior, and consequently conclude that both NIEPs and DB were present in our chromatographic eluates. We particularly analyzed the chromatographic behavior of exosomes derived from MRC-5 cells. Based on chromatograms obtained in ion-exchange mode, as well in hydrophobic interaction (results not shown), it can be concluded that the chromatographic behavior of exosomes (supernatant of non-infected MRC-5) and suspensions containing exosomes and viruses (supernatant of virus-infected MRC-5) are very similar and that they cannot be separated using these methods. The only method that theoretically could provide pure virus particles is immunoaffinity chromatography, ideally with a monoclonal antibody directed against virus surface proteins. Generally, one could expect problems with the stability of viruses in the harsh conditions required for affinity elution, but one possible solution is the native elution described previously [17]. 

## 5. Conclusions

We have developed scalable production of HCMV in serum-free conditions, followed by an anion-exchange chromatographic procedure for efficient purification of HCMV from the majority of host-cell DNA and most of the host-cell proteins that concentrates infective HCMV with excellent recoveries. We have also shown that MCMV is more robust and easier to handle compared to HCMV, and it shares the same chromatographic behavior as HCMV. These results provide important data for research on all upstream and downstream processes on these two viruses regarding biotechnological production and basic research.

## Figures and Tables

**Table 1:** Virus particle sizes, total particle recoveries, and virus infectivity recoveries after clarification by microfiltration through a 0.45 µm PVDF membrane and low speed centrifugation. Infectivity is expressed in comparison to the untreated, crude virus sample. Values of the MCMV filtered sample are expressed in comparison to the same sample clarified by centrifugation. Numbers in parentheses indicate the number of experiments performed.

			Mean/nm	Mode/nm	D90/nm	Size Ratio (%)	Particle Recovery (%)	Infectivety Recovery (%)
HCMV (*n* = 24)	microfiltration 0.45 µm	prior	224.5 ± 27.20	200.7 ± 31.85	322.7 ± 52.60	100	100 ± 22.2	76 ± 54.5
		post	226.1 ± 23.84	197.0 ± 26.44	322 ± 52.32			
MRC-5 exosomes	microfiltration 0.45 µm	prior	216 ± 12.42	163.5 ± 15.56	321,7 ± 17.72	100	116 ± 13.7	
		post	218.6 ± 6.13	163.0 ± 20.21	315.5 ± 9.51			
MCMV (*n* = 10)	centrifugation 3220× g; 7 min	prior	196.51 ± 10.8	155.8 ± 33.9	289.55 ± 15.1	100	95.72 ± 1.35	85 ± 17.3
		post	207.62 ± 2.2	138.6 ± 10.5	307.2 ± 7.1			
	microfiltration 0.45 µm	prior	207.62 ± 2.2	138.6 ± 10.5	307.2 ± 7.1	94	156 ± 2	94 ± 28.3
		post	195.9 ± 20.2	131.7 ± 15.9	288.8 ± 26.1			
M2-10B4 exosomes	microfiltration 0.45 µm	prior	189.3	140.4	266.9	101	90.71	
		post	191.6	133	292.3			

**Table 2:** Ultracentrifugation of HCMV and MCMV infected cell culture supernatants. Results of NTA measurements and CCID_50_ assays of the ultracentrifugated fractions, supernatants, and pellets are presented as the average ± standard deviation. Number of experiments *n* = 3.

	Infectivity Recovery (%)	Particle Recovery (%)	Size Ratio (%)	Infectivity Recovery (%)	Particle Recovery (%)	Size Ratio (%)	Infectivity Recovery (%)	Particle Recovery (%)	Size Ratio (%)
HCMV	1 h			2 h			4 h		
supernatant	0.2 ± 0.27	9 ± 3.6	63 ± 1.9	0	7 ± 4.4	77 ± 12.1	0	8 ± 8.9	74 ± 18.3
pellet	0.8 ± 1.13	59 ± 16.3	85 ± 8.8	7,6 ± 11.83	100 ± 81.6	99 ± 15.8	0.1 ± 0.23	63 ± 24.6	99 ± 7.1
MCMV	1 h			2 h			4 h		
supernatant	8.1 ± 3.1	9.682 ± 2.01	76.13 ± 1.53		ND		0	1.62 ± 0.81	75.52 ± 4.7
pellet	220.87 ± 121.44	95.21 ± 18.68	96.52 ± 4.41				28.22 ± 4.6	71.35 ± 1.72	105.41 ± 3.4

**Table 3:** Total particles measured by NTA and virus infectivity measured by CCID_50_ assay of HCMV purification using anion exchange chromatography on a QA monolith column. Number of experiments *n* = 5.

Binding Buffer	Elution Buffer (EB)	Chromatographic Fractions	EB (%)	Elution Molarity (M)	Particle Recovery (%)	Size Ratio (%)	Infectivity Recovery (%)
50 mM MES 0.15M NaCl pH 7.3	50 mM MES 1M NaCl pH 7.3	FT			2 ± 2.62	59 ± 15.2	0 ± 0.4
		E1	50	0.57 M	36 ± 4.32	87 ± 9.8	59 ± 36.8
		E2	100	1 M	41 ± 6.9	80 ± 7	43 ± 22.2

**Table 4:** Separation of host cell proteins and genomic DNA using anion-exchange chromatography of HCMV. Total particles were measured by NTA and virus infectivity by CCID_50_ assay. Starting sample is the clarified harvest. Protein and DNA amount calculations were made relative to the starting sample. Number of experiments *n* = 3.

Binding Buffer	Elution Buffer (EB)	Chromatographic Fractions	EB (%)	Elution Molarity (M)	Particle Recovery (%)	Size Ratio (%)	Infectivity Recovery (%)	Host Cell Proteins Percentage of Starting Sample (%)	Host Cell DNA Percentage of Starting Sample (%)
50mM MES pH 7.3	50mM MES 2M NaCl pH 7.3	FT			15.5 ± 11.5	79.3 ± 14.5	4.8 ± 3.5	54.5 ± 9.5	0.45 ± 0.35
		E1	12.5	0.25 M	21.7 ± 8.55	91 ± 10.4	128 ± 101	10.5 ± 8.5	1.45 ± 0.05
		E2	25	0.5 M	16.3 ± 13.5	99.3 ± 9.5	8.4 ± 9.8	2.5 ± 0.5	152 ± 39.4
		E3	50	1 M	11.4 ± 3.5	98 ± 6.7	15.5 ± 21.6	2.5 ± 0.5	
		E4	100	2 M	1 ± 0.3	111.3 ± 3.4	0.03 ± 0.05	0	

**Table 5:** Total particles measured by NTA and virus infectivity measured by CCID_50_ assay in fractions from MCMV purification using anion exchange chromatography on a QA monolith column. Number of experiments *n* = 8.

Binding Buffer	Elution Buffer (EB)	Chromatographic Fractions	EB (%)	Elution Molarity (M)	Particle Recovery (%)	Size Ratio (%)	Infectivity Recovery (%)
50 mM MES 0.15 M NaCl pH 7.3	50 mM MES 2M NaCl pH7.3	FT			0.15 ± 0.2	99.4 ± 42	0.02 ± 0.01
		E1	12.5	0.32 M	36.7 ± 7.9	104.9 ± 6.2	47 ± 27.8
		E2	25	0.61 M	10.8 ± 1.8	100.3 ± 1.8	7.4 ± 3.3
		E3	50	1.075 M	13.9 ± 4.8	106.4 ± 6.1	5.8 ± 4
		E4	100	2 M	1.9 ± 0.7	134.9 ± 19.3	1.4 ± 2

## Data Availability

Not applicable.
